# One-year surgical outcomes of the PreserFlo MicroShunt in glaucoma: a multicentre analysis

**DOI:** 10.1136/bjophthalmol-2021-320631

**Published:** 2022-04-01

**Authors:** Alexander Tanner, Fadi Haddad, Julia Fajardo-Sanchez, Ethan Nguyen, Kai Xin Thong, Sarah Ah-Moye, Nicole Perl, Mohammed Abu-Bakra, Avinash Kulkarni, Sameer Trikha, Gerassimos Lascaratos, Miles Parnell, Obeda Kailani, Anthony J King, Pavi Agrawal, Richard Stead, Konstantinos Giannouladis, Ian Rodrigues, Saurabh Goyal, Pirro G Hysi, Sheng Lim, Cynthia Yu-Wai-Man

**Affiliations:** 1 Faculty of Life Sciences & Medicine, King's College London, London, UK; 2 Department of Ophthalmology, Guy’s and St Thomas’ NHS Foundation Trust, London, UK; 3 Department of Ophthalmology, King's College Hospital NHS Foundation Trust, London, UK; 4 Department of Ophthalmology, Nottingham University Hospitals NHS Foundation Trust, Nottingham, UK

**Keywords:** Glaucoma, Intraocular pressure, Treatment Surgery, Wound healing

## Abstract

**Background/aims:**

To evaluate the efficacy and safety of the PreserFlo MicroShunt glaucoma device in a multicentre cohort study.

**Methods:**

All consecutive patients who received the microshunt with mitomycin-C (MMC) 0.4 mg/mL from May 2019 to September 2020 in three UK tertiary centres. Primary outcome at 1 year was a complete success, with failure defined as intraocular pressure (IOP) >21 mmHg or <20% reduction, IOP≤5 mmHg with any decreased vision on two consecutive visits, reoperation or loss of light perception vision. Secondary outcomes were IOP, best-corrected visual acuity, medications, complications, interventions and reoperations. We also performed subgroup analyses for severe glaucoma and assessed risk factors for failure.

**Results:**

104 eyes had 1-year follow-up. Complete and qualified success at 1 year were achieved in 51.9% (N=54) and 16.4% (N=17), respectively, and failure occurred in 31.7% (N=33). There was a significant reduction in IOP (mmHg) from preoperatively (23.4±0.8, N=104) to 12 months (14.7±0.6, N=104) (p<0.0001). Antiglaucoma medications also decreased from preoperatively (3.4±0.1, N=104) to 12 months (0.7±0.1, N=104) (p<0.0001). Multivariate analyses showed an association between higher mean deviation and failure (HR 1.055, 95% CI 1.0075 to 1.11, p=0.0227). Complications were hypotony (19.2%; N=20), choroidal detachments (10.6%; N=11), hyphaema (5.8%; N=6) and bleb leak (5.8%; N=6). Needling and 5-fluorouracil injections were performed in 12.5% (N=13) and 33.7% (N=35), respectively, and 11.5% (N=12) required revision surgery.

**Conclusion:**

The PreserFlo MicroShunt with MMC 0.4 mg/mL showed an overall success rate of 68.3% at 1 year, and led to significant IOP and medication reduction with a low rate of adverse effects.

**Key message**

**What is already known on this topic**

- The recently published randomised clinical trial using a lower dose of mitomycin-C (MMC) of 0.2 mg/mL for 2 min has shown a significantly lower success rate and higher mean intraocular pressure (IOP) and number of medications at 1 year compared with trabeculectomy in POAG patients. However, the results may not reflect the outcomes associated with the higher concentration of MMC 0.4 mg/mL, which is routinely used in standard of care.

**What this study adds**

- This study evaluates the real-world efficacy and safety of the PreserFlo MicroShunt from a multicentre perspective, based on three tertiary referral centres in the UK, and using the MMC concentration of 0.4 mg/mL in different types of glaucoma. Real-world multicentre experience of the PreserFlo MicroShunt with MMC 0.4 mg/mL shows an overall success rate of 68.3% at 1 year, 37% reduction in IOP, 79% reduction in antiglaucoma medications, and a good safety profile.

**How this study might affect research, practice or policy**

- This study contributes real-world experience of the PreserFlo minimally invasive glaucoma surgery device, which allows more generalisability to everyday practice than trial-reported data, is consistent with the previously reported real-world outcomes for trabeculectomy in the UK, and that would help in clinical decision making in glaucoma surgery.

## Introduction

Glaucoma is the leading cause of irreversible blindness worldwide, currently affecting 76 million people and its prevalence is estimated to increase to 112 million by 2040.^1^ Treatment involves reducing intraocular pressure (IOP) with medications, laser or surgery. First described in 1968, trabeculectomy remains the mainstay of surgical treatment for medically uncontrolled glaucoma.^2^ Despite its efficacy at lowering IOP, trabeculectomy requires significant postoperative management, which delays recovery, and is associated with potentially sight-threatening complications.^3^

The PreserFlo MicroShunt (Santen, Miami, Florida, USA) offers a minimally invasive glaucoma surgery (MIGS) alternative to trabeculectomy. It is a device measuring 8.5 mm long with a 350 µm outer diameter and 70 µm lumen, and is made from a biocompatible material: poly(styrene-block-isobutylene-block-styrene), alternatively known as ‘SIBS’.^4 5^ Like trabeculectomy, the PreserFlo MicroShunt drains aqueous humour from the anterior chamber to a bleb in the subconjunctival space. However, the procedure is considered to be less invasive and is associated with a lower risk of complications and faster recovery. The risk of hypotony is minimised in the PreserFlo MicroShunt by a valve-less intrinsic flow limiting design. The biocompatible material has also been designed to decrease postoperative inflammation and the risk of fibrosis.^6^

There have been a few studies that have investigated the effectiveness of the PreserFlo MicroShunt. These studies have reported reasonable success rates,^7–11^ but the recently published randomised clinical trial using a lower dose of mitomycin-C (MMC) of 0.2 mg/mL for 2 min has shown a significantly lower success rate and higher mean IOP and number of medications at 1 year compared with trabeculectomy.^12^ However, the results may not reflect the outcomes associated with the higher concentration of MMC 0.4 mg/mL, which is routinely used in standard of care.^7 8 11 13^ The recent randomised controlled trial (RCT) also studied only primary open angle glaucoma (POAG) patients whereas we included other types of glaucoma (e.g., primary angle closure glaucoma (PACG), secondary OAG) in our study. Our study thus aims to evaluate the real-world efficacy and safety of the PreserFlo MicroShunt from a multicentre perspective, based on three tertiary referral centres in the UK, and using the MMC concentration of 0.4 mg/mL.

## Materials and methods

### Study design

This is a multicentre cohort study of all consecutive patients who received the PreserFlo Microshunt by 14 fellowship-trained glaucoma consultants in three tertiary referral centres from May 2019 to September 2020: Guy’s and St Thomas’ NHS Foundation Trust, King’s College Hospital NHS Foundation Trust, and Nottingham University Hospitals NHS Foundation Trust. Data were obtained from electronic and written medical records. Identifiable patient data were anonymised and categorised in excel spreadsheets.

### Inclusion and exclusion criteria

We included all patients aged over 18 years with a previous diagnosis of glaucoma, having an IOP above target despite maximal medical treatment, and receiving the PreserFlo MicroShunt with intraoperative MMC 0.4 mg/mL. Following the World Glaucoma Association guidelines, we have excluded all the second eye surgeries of patients who had microshunt surgery in both eyes during the study period.^14^

### Ab externo microshunt surgery

After placing a corneal traction suture, a conjunctival and Tenon’s capsule peritomy was made at the superior limbus. Dissection of both layers was performed posteriorly and haemostasis was achieved using diathermy. MMC 0.4 mg/mL was applied between 2 and 5 min onto the bare sclera and inside the conjunctival flap by placing three identical sponges (provided as standard in the microshunt pack) soaked in MMC, followed by copious irrigation with balanced salt solution. The sclera was then marked 3 mm posterior to the limbus and a superficial scleral pocket was made using the blade provided in the microshunt pack. Using a 25 G orange needle, a tunnel was created through the scleral pocket and into the anterior chamber. The microshunt was then inserted inside the tunnel, leaving approximately 2–3 mm of the tube inside the anterior chamber and allowing the wings of the device to rest inside the scleral pocket. Finally, flow through the device was confirmed by the presence of drop formation at the scleral end of the microshunt before Tenon’s and conjunctival closure.

### Data collection

Demographic data, including age, gender and ethnicity, and any previous ocular surgeries and laser procedures were collected from the medical records. Detailed clinical data, including type of glaucoma, best-corrected visual acuity (BCVA), IOP, central corneal thickness (CCT), cup-to-disc ratio (C/D) and antiglaucoma medications (topical and oral) were recorded preoperatively and at each postoperative visit (day 1, week 1, month 1, month 3, month 6, month 12). Visual field testing was carried out using the Humphrey Field Analyser Mark II SITA Fast 24–2 (Carl Zeiss, Birmingham, UK). Optical coherence tomography (OCT) imaging of the disc was also performed using the OCT-Spectralis (Heidelberg Engineering Inc., Massachusetts, USA) or the DRI-OCT Triton machine (Topcon, Newbury, UK).

The grade of the surgeon, any intraoperative complications, and the length of time and concentration of intraoperative MMC application were recorded. Detailed early (<3 months) and late (>3 months) postoperative complications, including hypotony, choroidal detachments, hyphaema, bleb leak, microshunt exposure, as well as postoperative interventions, including bleb injections and needling, were collected. We also recorded early (<3 months) and late (>3 months) postoperative revision surgeries as well as reoperations with another type of glaucoma surgery.

### Outcome measures

Primary outcome at 1 year was defined as a complete success (CS), qualified success (QS), or failure (F).^15^ CS was defined as: IOP of 6–21 mmHg (inclusive) with ≥20% reduction from preoperative IOP without anti-glaucoma medications. QS was defined as the same parameters as CS but with antiglaucoma medications. F was defined as IOP >21 mmHg or not reduced by 20%; IOP ≤5 mmHg with any decreased vision on two consecutive visits; reoperation for glaucoma; or loss of light perception vision. We also analysed the causes for any treatment failure at 1 year, and recorded the number of anti-glaucoma drugs that were being used in each cause of treatment failure. As some patients require lower IOP control, we further examined the CS, QS and F for IOP of 6–17 mmHg and 6–14 mmHg.

Secondary outcomes at 1 year were IOP, BCVA, antiglaucoma medications, intraoperative and postoperative complications, postoperative interventions, revisions and reoperations. We defined the severity of the glaucoma according to the extent of visual field loss (Hodapp-Parrish-Anderson classification), and performed subgroup analyses for the patients with severe/advanced glaucoma.^16^

### Statistical analyses

All graphs display mean and standard error of the mean (SEM). Statistical analysis was performed using the Student’s t-test to calculate statistically significant differences and p values. Survival analyses for CS and QS were performed using the Kaplan-Meier log rank test. The time to event analyses were conducted using the statistical package ‘survival’ and the results were visualised using the package ‘*survminer*’ in R V.4.02 (https://cran.r-project.org/). Statistically significant differences were expressed as *p<0.05; ** p<0.01; ***p<0.001.

## Results

### Baseline characteristics

We identified 104 consecutive patients that had the PreserFlo MicroShunt with MMC 0.4 mg/mL in all three UK tertiary centres from May 2019 to September 2020. Baseline characteristics are detailed in table 1. The mean age was 68.9±1.0 years (SEM) and 50.9% (N=53) were female. Most patients were white Caucasian (56.7%, N=59) and Afro-Caribbean (37.5%, N=39). The most common diagnoses were primary open angle glaucoma in 73.1% (N=76), secondary open angle glaucoma in 11.5% (N=12), and primary angle closure glaucoma in 5.8% (N=6).

**Table 1:** Baseline characteristics

**Demographics**	**Total (N=104 patients**)
Age in years, mean (range, SEM)	68.9 (60–81, 1.0)
Female, no (%)	53 (50.9)
Ethnicity, no (%)	
White Caucausian, no (%)	59 (56.7)
Other Ethnicities, no (%)	45 (43.3)
Afro-Caribbean	39 (37.5)
Asian	5 (4.8)
Hispanic Latino	1 (1.0)
Preoperative clinical characteristics	Total (N=104 eyes)
Left eye, no (%)	69 (66.6)
Preoperative BCVA (logMAR), mean (range, SEM)	0.3 (−0.2 to 2.0, 0.02)
Preoperative BCVA 0.4 logMAR or worse, mean (range, SEM)	0.50 (0.5–1.25, 0.02)
IOP, mean (range, SEM)	23.6 (14–50, 0.5)
CCT, mean (range, SEM)	536.3 (418–664, 3.6)
C/D Ratio, mean (range, SEM)	0.85 (0.3–1.0, 0.01)
No of medication classes, mean (range, SEM)	3.0 (0–5, 0.1)
Visual field MD, no (%), mean (range, SEM)	100 (96.2), –14.8 (+1.52 to −33.9, 0.6)
Mild (MD>-6 dB), no (%), mean (range)	17 (16.3), –3.0 (+1.52 to −5.3)
Moderate (MD=-6 to −12 dB), no (%), mean (range)	21 (20.2), –8.7 (-6.3 to −11.4)
Severe/advanced (MD<-12 dB), no (%), mean (range)	62 (59.6), –21.7 (-12.9 to −33.9)
OCT disc RNFL thickness (microns), no (%), mean (range, SEM)	83 (79.8), 63.3 (30–124, 1.5)
Previous ocular surgery	
Trabeculectomy, no (%)	12 (11.5)
Baerveldt tube, no (%)	1 (1.0)
Selective laser trabeculoplasty (SLT), no (%)	12 (11.5)
Cyclodiode, no (%)	6 (5.8)
iStent inject (±combined surgery), no (%)	10 (9.6)
iStent inject only	*6* (*5.8*)
iStent inject (+phacoemulsification)	*4* (*3.8*)
Xen, no (%)	2 (1.9)
Kahook dual blade, no (%)	1 (1.0)
Phacoemulsification, no (%)	31 (29.8)
Pars plana vitrectomy (PPV), no (%)	4 (3.8)
Laser-assisted in situ keratomileusis (LASIK), no (%)	1 (1.0)
No previous surgery, no (%)	35 (33.7)
Diagnosis, Primary open angle glaucoma (POAG), no (%)	**76** (**73.1**)
Diagnosis, no POAG, no (%)	28 (26.9)
Secondary open angle glaucoma	**12** (**11.5**)
Pigmentary	*4* (*3.8*)
Uveitic	*2* (*1.9*)
Pseudoexfoliation	*5* (*4.8*)
Traumatic	*1* (*1.0*)
Primary angle closure glaucoma	**6 (5.8)**
Secondary angle closure glaucoma	**6 (5.8)**
Neovascular glaucoma	*5* (*4.8*)
Plateau Iris	*1* (*1.0*)
Normal tension glaucoma	**4** (**3.8**)
Intraoperative MMC 0.4 mg/mL, no (%)	
5 min	22 (21.2)
4 min	33 (31.7)
3 min	47 (45.2)
2 min	2 (1.9)
Grade of surgeon, no (%)	
Consultant	81 (77.9)
Fellow	23 (22.1)

BCVA, best-corrected visual acuity; CCT, central corneal thickness; C/D, cup-to-disc; IOP, intraocular pressure; logMAR, logarithm of the minimum angle of resolution; MD, mean deviation; MMC, mitomycin-C; OCT, optical coherence tomography; RNFL, retinal nerve fibre layer.

Most surgeries were performed by consultants (77.9%, n=81), with the remainder being done by post-CCT glaucoma fellows (22.1%, n=23). All patients received intraoperative MMC 0.4 mg/mL between 2 and 5 min. The mean preoperative best-corrected visual acuity BCVA (logMAR, Logarithm of the Minimum Angle of Resolution) was 0.30±0.02, with 22 eyes (21.2%) recording 0.4 logMAR or worse and the mean BCVA in this subgroup was 0.50±0.02. At baseline, mean IOP was 23.6±0.5 mmHg and C/D ratio was 0.85±0.01. Most patients were on multiple antiglaucoma medications preoperatively with a mean of 3.0±0.1. Visual field tests were available in 100 eyes (96.2%) and the average mean deviation (MD) was −14.8±0.6. We further classified the eyes as having mild (MD ≥6 dB, 16.3%, n=17), moderate (MD between −6 to −12 dB, 20.2%, n=21), or severe/advanced glaucoma (MD ≤12 dB, 59.6%, n=62). OCT disc assessments were also available in 83 eyes (79.8%) and the mean RNFL thickness was 63.3±1.5 µm.

Out of the 104 eyes, 69 eyes (66.3%) had undergone previous ocular surgery (table 1). Trabeculectomy was performed in 12 eyes (11.5%) and a baerveldt tube was inserted in one eye (1.0%). Selective laser trabeculoplasty was performed in 12 eyes (11.5%) while cyclodiode laser was carried out in six eyes (5.8%). MIGS was also performed in 13 eyes (12.5%). Most of these were iStent inject (9.6%, N=10), of which 4 eyes (3.8%) underwent combined Phaco+iStent inject surgery. At baseline, 29.8% (N=31) were pseudophakic.

### Primary outcome: surgical success at 1 year

**Figure 1:**
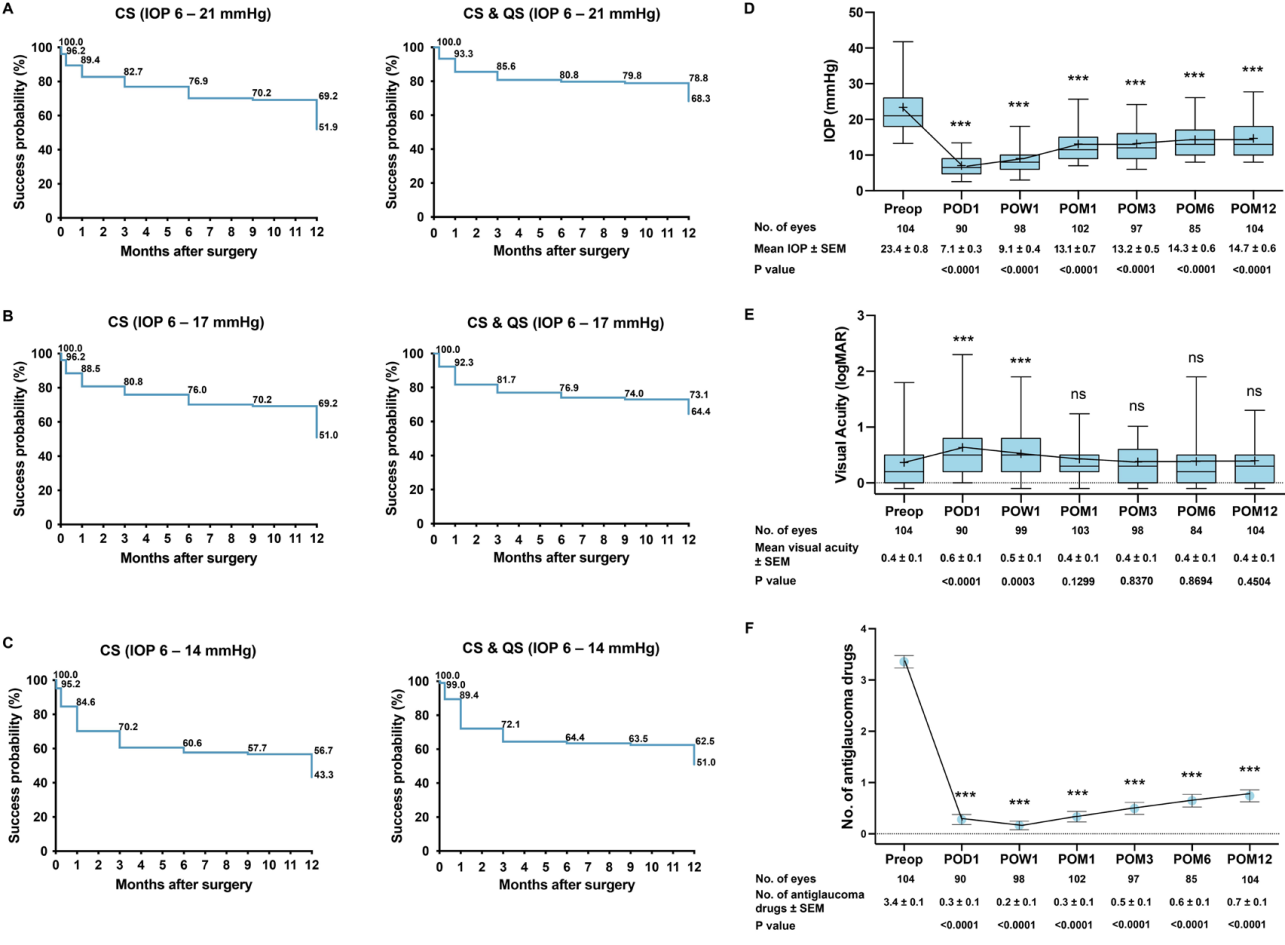
Kaplan-Meier survival curves over 12 months for complete success (CS) and combined CS and qualified success (CS & QS) for: (A) IOP 6–21 mmHg; (B) IOP 6–17 mmHg; (C) IOP 6–14 mmHg. (D) Preoperative and postoperative IOP over 12 months. Box and whisker plot with line joining the mean at each time point. The middle line indicates the median, the lower and upper ends of boxes indicate the 5–95 percentiles. (E) Preoperative and postoperative logMAR best-corrected visual acuity over 12 months. (F) Postoperative change in the number of antiglaucoma medications over 12 months compared with preoperatively. 14 eyes of the 104 eyes that had 1 year follow-up data also had reoperations for glaucoma, and we censored the IOP, BCVA and medication data at the time of the second glaucoma operation. Mean±SEM. ***p<0.001. BCVA, best-corrected visual acuity; logMAR, Logarithm of the Minimum Angle of Resolution; IOP, intraocular pressure; ns, not significant; POD, postoperative day; POW, postoperative week; POM, postoperative month.

A total of 104 eyes had 1-year follow-up data. CS and QS at 1 year for IOP 6–21 mmHg were achieved in 51.9% (N=54) and 16.4% (N=17), respectively, and F occurred in 31.7% (N=33) (table 2A). Kaplan-Meier survival probability (%) for CS for IOP 6–21 mmHg was 96.2, 89.4, 82.7, 76.9, 70.2, 69.2 and 51.9 at 1 day, 1 week, 1 month, 3 months, 6 months, 9 months and 12 months, respectively (figure 1A). Survival probability (%) for combined CS+QS for IOP 6–21 mmHg was 100.0, 93.3, 85.6, 80.8, 79.8, 78.8 and 68.3 at 1 day, 1 week, 1 month, 3 months, 6 months, 9 months and 12 months, respectively (figure 1A). We further examined the CS, QS and F for IOP 6–17 mmHg and 6–14 mmHg. CS and QS at 1 year for IOP 6–17 mmHg were achieved in 51.0% (N=53) and 13.4% (N=14), respectively, and F occurred in 35.6% (N=37) (figure 1B). CS and QS at 1 year for IOP 6–14 mmHg were achieved in 43.3% (N=45) and 7.7% (n=8), respectively and F occurred in 49.0% (N=51) (figure 1C).

**Table 2:** (A) Primary outcome for IOP 6–21 mmHg at 1 year; (B) reasons for treatment failure

(A) Primary outcome	No of eyes (%)
Complete success	54 (51.9)
Qualified success	17 (16.4)
Failure	33 (31.7)
**(B) Reasons for failure**	**Total no (%**)
IOP >21 mmHg	14 (13.5)
<20% IOP reduction	24 (23.1)
IOP ≤5 mmHg with vision loss on two consecutive visits	0
Reoperation for glaucoma	14 (13.5)
Loss of light perception vision	0

IOP, intraocular pressure.

The reasons for treatment failure at 1 year are detailed in table 2B. We also analysed the number of antiglaucoma medications by the cause of treatment failure at 1 year (online supplemental table 1). Of the 33 eyes that failed surgery for IOP 6–21 mmHg, the most common reason was an IOP reduction less than 20% (compared with baseline) in 24 eyes (23.1%). Another common reason for treatment failure at 1 year was an IOP over 21 mmHg in 14 eyes (13.5%). Out of the 18 eyes that failed from pressure-related criteria alone (IOP >21 mmHg or IOP reduction <20% or both), 10 eyes were on no drops, 5 eyes were on a single agent, 2 eyes were on dual therapy, and only one eye was on three agents. Preoperatively, they were on an average of 3.2 anti-glaucoma medications, and their mean IOP was 19.6 mmHg preoperatively and 19.3 mmHg postoperatively. It is interesting to note that 83.3% of the eyes that failed at 1 year for pressure-related criteria alone were on a single anti-glaucoma medication or less.

10.1136/bjophthalmol-2021-320631.supp1 Supplementary data

In addition, reoperation for glaucoma was the cause of treatment failure at 1 year in 14 eyes (13.5%) (table 2B). No patients failed due to loss of light perception vision, or low IOP ≤5 mmHg with any decreased vision on two consecutive visits.

### Secondary outcomes and factors associated with failure

Fourteen eyes of the 104 eyes that had 1 year follow-up data also had reoperations for glaucoma, and we censored the IOP, BCVA and medication data at the time of the second glaucoma operation. There was a significant decrease in IOP (mmHg) from preoperatively (23.4±0.8, N=104) to 1 day (7.1±0.3, N=90), 1 week (9.1±0.4, N=98), 1 month (13.1±0.7, N=102), 3 months (13.2±0.5, N=97), 6 months (14.3±0.6, N=85), and 12 months (14.7±0.6, N=104) (p<0.0001 for all time points) (figure 1D). BCVA decreased from preoperatively (0.4±0.1, N=104) to 1 day (0.6±0.1, N=90) (p<0.0001) and 1 week (0.5±0.1, N=99) (p=0.0003), but went back to the preoperative level thereafter: 1 month (0.4±0.1, N=103) (p=0.1299), 3 months (0.4±0.1, N=98) (p=0.8370), 6 months (0.4±0.1, N=84) (p=0.8694) and 12 months (0.4±0.1, N=104) (p=0.4504) (figure 1E). The number of antiglaucoma medications also significantly decreased from preoperatively (3.4±0.1, N=104) to 1 day (0.3±0.1, N=90), 1 week (0.2±0.1, n=98), 1 month (0.3±0.1, N=102), 3 months (0.5±0.1, N=97), 6 months (0.6±0.1, N=85), and 12 months (0.7±0.1, N=104) (p<0.0001 for all time points) (figure 1F).

We also performed a subgroup analysis of the 62 eyes (59.6%) with severe/advanced glaucoma (MD ≤12 dB). CS was similar in the severe/advanced glaucoma eyes (50.0%, N=31) and the mild-moderate glaucoma eyes (52.6%, N=20). However, QS was significantly higher in the severe/advanced glaucoma eyes (24.2%, N=15) compared with the mild-moderate glaucoma eyes (2.6%, N=1), leading to a lower failure rate in the severe/advanced glaucoma eyes (25.8%, N=16) compared with the mild-moderate glaucoma eyes (44.8%, N=17).

**Figure 2:**
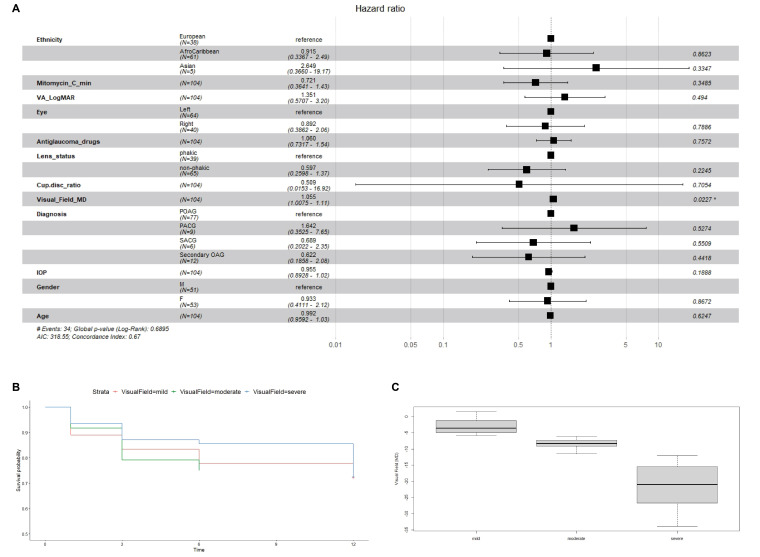
(A) HRs with 95% CI and p values for various factors potentially associated with failure: ethnicity, mitomycin-C treatment time (minutes), baseline best-corrected visual acuity (VA), side of operated eye, number of preoperative antiglaucoma drugs, lens status, preoperative cup-disc ratio, preoperative mean deviation (MD), type of glaucoma (POAG, PACG, secondary OAG, secondary ACG), preoperative IOP (mmHg), gender, age. (B) Kaplan-Meier survival curves over 12 months according to the severity of glaucoma: mild (MD≥6 dB), moderate (MD = −6 to −12 dB), severe/advanced severity (MD ≤12 dB). (C) Box and whisker plots showing distribution of MD for the mild, moderate and severe/advanced glaucoma groups. IOP, intraocular pressure; POAG, primary open angle glaucoma; PACG, primary angle closure glaucoma; SACG, secondary angle closure glaucoma; Secondary OAG, secondary open angle glaucoma.

We further assessed the hazard ratios (HRs) for various factors potentially associated with failure: ethnicity, MMC treatment time (minutes), baseline BCVA, side of operated eye, number of preoperative anti-glaucoma drugs, lens status, preoperative cup-disc ratio, preoperative MD, type of glaucoma, preoperative IOP (mmHg), gender and age (figure 2A). Multivariate analyses showed that high MD was associated with a higher risk of failure (HR 1.055, 95% CI 1.0075 to 1.11, p=0.0227). There were no statistically significant associations found between failure and the other factors. Using a univariate model to study MD alone, we found a similar trend for severe/advanced glaucoma (MD ≤12 dB) to be associated with a lower risk of failure (figure 2B, C).

### Intraoperative and postoperative complications

Intraoperative, early (<3 months) and late (>3 months) postoperative complications are detailed in table 3A. Intraoperative complications included five eyes (4.8%) with conjunctival buttonholing, of which one eye developed a bleb leak at 1 week that was successfully sutured in theatre at 3 weeks. This patient’s bleb was still functioning at 1 year without the need for antiglaucoma medications. Postoperatively, bleb leaks occurred early within 1 week of surgery in six eyes (5.8%), with two eyes needing early revision and four eyes resolving spontaneously after conservative management. One patient with a persistent bleb leak underwent multiple surgeries to address a significantly thin and avascular conjunctiva from previous trabeculectomy surgery, and the microshunt was subsequently removed at 18 months.

**Table 3:** (A) Intraoperative and postoperative complications; (B) postoperative interventions; (C) revisions, reoperations and other surgeries

(A) Complications	Intraoperative	Early postoperative (<3 months)	Late postoperative (>3 months)
Conjunctival buttonhole	5 (4.8%)		
Repeated passes to insert microshunt	5 (4.8%)		
Hypotony (IOP ≤5 mmHg)		20 (19.2%)	1 (1.0%)
Numerical hypotony without sequelae		8 (7.7%)	0
Clinical hypotony with sequelae		12 (11.5%)	1 (1.0%)
Hypotonous maculopathy		1 (1.0%)	0
Choroidal detachment		11 (10.6%)	0
Iridocorneal touch		3 (2.9%)	0
Hyphaema/microhyphaema		6 (5.8%)	0
Bleb leak		6 (5.8%)	0
Corneal oedema		1 (1.0%)	0
Corneal abrasion		1 (1.0%)	0
Microshunt exposure		0	1 (1.0%)
Cystoid macular oedema		0	1 (1.0%)

(B) Interventions	No of eyes (%)
5-Fluorouracil (5FU) injection	35 (33.7)
Dexamethasone/ betamethasone injection	24 (23.1)
Combined 5FU+steroid injection	12 (11.5)
Tissue plasminogen activator injection	1 (1.0)
Bevacizumab injection	1 (1.0)
Any subconjunctival injection	46 (44.2)
Repeated subconjunctival injections (>1 injection)	25 (24.0)
Viscoelastic injection in anterior chamber	6 (5.8)
Needling	13 (12.5)
Repeated needling (>1 needling)	7 (6.7)

(C) Reoperations	Early postoperative (<3 months)	Late postoperative (>3 months)
Bleb revision for high IOP	1 (1.0%)	8 (7.7%)
Bleb revision for leak	2 (1.9%)	1 (1.0%)
Bleb revision for exposed tube	0	1 (1.0%)
Glaucoma drainage device	0	1 (1.0%)
iStent inject	0	1 (1.0%)
Phacoemulsification	0	2 (1.9%)

IOP, intraocular pressure.

Clinical hypotony was defined as IOP ≤5 mmHg with sequelae, for example, shallow anterior chamber, hypotonous maculopathy, or choroidal detachment. Numerical hypotony was defined as IOP ≤5 mmHg without any sequelae. Early postoperative hypotony was encountered in 20 eyes (19.2%), where all but 1 case (1.0%) resolved within 3 months. Eight eyes (7.7%) only experienced numerical hypotony with no adverse sequelae. The eye with persistent late hypotony was due to the ongoing bleb leak described above. In the early postoperative period, hypotony led to maculopathy in 1 eye (1.0%), choroidal detachments in 11 eyes (10.6%), and iridocorneal touch in 3 eyes (2.9%). Tube migration was observed in one eye (1.0%) with hypotony, where the wings had become visible under the conjunctiva from posterior dislodgement, and the tube was reinserted into place at day 1.

Of the 104 eyes, 32 eyes (30.8%) lost ≥2 Snellen lines of visual acuity and 23 eyes (22.1%) lost ≥3 Snellen lines. Hyphaema/microhyphaema occurred early postoperatively in six eyes (5.8%) and all resolved within 3 months. Corneal complications were present in two eyes, with corneal oedema and corneal abrasion recorded in one eye (1.0%) each. All cases were managed medically with resolution by 3 months, at which time central visual acuity was not affected. Late tube exposure also occurred in one eye (1.0%) after 3 months and required surgical revision. There were no cases of endophthalmitis reported.

### Postoperative interventions

The most common interventions performed postoperatively were subconjunctival injections of 5-FU or steroid (dexamethasone/betamethasone) in 35 eyes (33.7%) and 24 eyes (23.1%), respectively (table 3B). Subconjunctival bevacizumab injection was given in one eye (1.0%). Overall, 46 eyes (44.2%) needed a subconjunctival injection of at least one agent, and 25 eyes (24.0%) needed more than 1 subconjunctival injection (range: 2–5). Injection of a viscoelastic in the anterior chamber was also performed in six eyes (5.8%) with hypotony: two eyes due to hypotonous maculopathy, two eyes due to iridocorneal touch, and the remaining two were performed for a shallow anterior chamber and choroidal folds.

Needling was performed in 13 eyes (12.5%), of which 7 eyes (6.7%) required repeated needling. Most needling interventions were performed in clinic but 3 eyes required needling in theatre. Of the 13 eyes needled, 8 eyes (61.5%) failed at 12 months, 4 eyes (30.8%) reached QS and only 1 eye (7.7%) achieved CS. Overall, from the 13 eyes that required a needling intervention, 8 eyes (61.5%) went on to have surgical revision in theatre.

Microshunts may require less intensive early post-operative management. It is interesting to note that of the 90 eyes reviewed at day 1 in our study, only two eyes (2.2%) needed an intervention. One eye had a 5-fluorouracil (5FU) injection and the other eye required the reinsertion of the microshunt tube into the scleral tunnel. The remaining 88 eyes (97.8%) did not require any intervention at day 1 after microshunt surgery.

### Revisions and reoperations

Table 3C shows the reoperations and subsequent surgeries in the early and late postoperative periods. Ab externo bleb revision occurred in 12 eyes (11.5%), with 3 eyes (2.9%) occurring within the first 3 months. Of these, two eyes (1.9%) were for bleb leak and one eye (1.0%) for early tube obstruction. One eye had a persistent bleb leak as previously mentioned and was recorded again in the late postoperative period. Excluding this, there were nine cases (8.7%) of late revisions, of which eight eyes (7.7%) required surgery due to bleb scarring from either a flat or encapsulated bleb; although more specifically in one eye, the tube had become obstructed. One eye (1.0%) required bleb revision for tube exposure.

Further glaucoma surgery was recorded in two eyes (1.9%). One eye underwent Ahmed glaucoma drainage device insertion, and the previous microshunt was exposed and partially revised at the same time to counter the anticipated postoperative hypertensive phase. Another eye with a scarred bleb also underwent combined Phaco+iStent inject surgery. A total of two eyes (1.9%) underwent subsequent cataract surgery, all in the late postoperative period and combined with 5FU injection into the bleb.

## Discussion

The aim of this multicentre study was to investigate the real-world experience of the PreserFlo MIGS device. This allows more generalisability to everyday practice than trial-reported data and is consistent with the previously reported real-world outcomes for trabeculectomy in the UK.^17^ At 1 year using our study failure criteria, we observed complete and qualified success in 51.9% and 16.4% of eyes, respectively, and failure in 31.7%.

Our results are similar to those of the recently published prospective multicentre RCT comparing the effectiveness and safety of stand-alone microshunt (N=395) to trabeculectomy (N=132) in mild-to-severe POAG patients (online supplemental table 2).^12^ The authors used a similar surgical success endpoint defined as ≥20% reduction in mean diurnal IOP from baseline at 1 year without increasing the number of anti-glaucoma medications. At 1 year, the success rate for the microshunt group (53.9%) was significantly lower compared with the trabeculectomy group (72.7%). After running an analysis of our data and applying the same criteria as used by the RCT in their post hoc analyses, we found that 67.3% of our patients achieved CS at 1 year compared with 65.1% in the RCT.

10.1136/bjophthalmol-2021-320631.supp2 Supplementary data

Interestingly, our study suggests that higher concentrations of MMC (0.4 mg/mL for 2–5 min) used in all patients in our study only slightly increased the success rate of microshunt surgery at 1 year compared with the RCT that used 0.2 mg/mL for 2 min. Another study also reported that MMC concentration might not affect overall success (78.1% for 0.2 mg/mL and 74.4% for 0.4 mg/mL), but that there was a trend towards lower IOP and higher medication reduction in the 0.4 mg/mL MMC subgroup.^13^ A potential contributing factor for the small increase in success rate in our study despite the higher concentration of MMC (0.4 vs 0.2 mg/mL) might be the significantly higher proportion of Afro-Caribbean patients in our cohort (37.5% Afro-Caribbean, 56.7% white Caucasian) compared with the patients in the RCT (18.0% Afro-Caribbean, 78.7% white Caucasian).

Durr *et al* also studied the efficacy of the SIBS microshunt in patients with refractory glaucoma (N=85), using the same IOP ranges as in our study (6–21 mmHg inclusive). The authors described CS in 61.0% and failure in 17.8% of eyes.^8^ Our results showed that there was a trend for severe/advanced glaucoma (MD ≤12 dB) to be associated with a lower risk of failure. Durr *et al* similarly found that patients with advanced glaucoma achieved greater success with microshunt surgery than those with mild-to-moderate glaucoma (adjusted HR 2.37; 95% CI 1.23 to 4.59). This might be due to factors that influence the outcomes of microshunt surgery in different types of glaucoma and disease severity, namely that clinicians might be more reluctant to leave patients with advanced glaucoma without drops after surgery so that less patients would fail from IOP lowering-related criteria. This is demonstrated in our study by the greater number of antiglaucoma medications prescribed on average at 12 months postoperatively in patients with advanced glaucoma (0.79) compared with patients with mild to moderate glaucoma (0.49).

Of the 18 eyes that failed due to pressure-related criteria alone, 83% were on a single agent or less at month 12, in fact, 56% were not taking any antiglaucoma medication at all. This leads us to propose that if medications had been reinstated when IOP began to rise, many of these cases could potentially have fallen under the criteria of QS, particularly as the mean postoperative IOP was 19.3 mmHg in this subgroup. As this was a retrospective study with no strict protocol as to when anti-glaucoma medications should be added postoperatively, this decision would have been left up to the discretion of the treating clinician who may have regarded the IOP at 1 year as satisfactory in the absence of any glaucoma progression. This is certainly plausible given that the average number of medications decreased from 3.2 preoperatively to 0.7 postoperatively in this subgroup. This is consistent with clinical decisions taken in the real-world setting and reflects routine standard of care.

IOP is a good surrogate marker for quantitative evaluation of disease control. We found that during the first year, following an initial decrease, there was a gentle upwards trend in IOP. There is a paucity of studies that enable direct comparison; yet those that do exist seem to corroborate our findings. A smaller observational study (N=23) that investigated the long-term efficacy and safety of the microshunt over a 3-year period described a similar trend in IOP.^7^ Around the 1-year mark, they found that IOP had stabilised (10.7±2.8 mmHg), remaining largely unchanged at 3 years (10.7±3.5 mmHg). Their mean IOP at 1 year was slightly lower than the one in our study (14.7±0.6 mmHg, N=104); however, owing to the low statistical power of this study (N=23), it is difficult to draw any conclusions from it. Nonetheless, it would be interesting to see whether the IOP stabilises in our cohort at 1 year or whether it continues to rise in subsequent years. Durr *et al* also described a similar trend in IOP.^8^ Although it is not directly comparable, their median IOP at 1 year was 13.0 mmHg (IQR 10.0–17.0, N=85), which is close to the mean IOP in our study. Likewise, a two-centre study that took place in France and the Dominican Republic demonstrated an IOP at 12 months that was within the same range as in our study (13.3±3.3 mmHg, N=87).^18^

Variation in the results of the microshunt studies may in part be due to the differences in demographics. Our population comprised 56.7% white Caucasian and 43.3% non-Caucasian, while the population in the study by Durr *et al*, comprised 43.5% Caucasian and 56.5% non-Caucasian.^8^ Moreover, 36.5% of the individuals in their study were female and 63.5% male, compared with our roughly equal split of females (50.9%) to males (49.1%). It has been reported in several studies that both race and gender have the potential to influence results owing to inherent characteristics that predispose to the development and progression of glaucoma.^19 20^ Another factor that may influence the results is the number and grade of the operating surgeons. The study by Durr *et al* was conducted at one centre by one consultant and the fellows, while our study was conducted in three tertiary centres and included data from 14 consultants and their fellows.^8^ While the use of one consultant helps to decrease data variability, it reduces the generalisability of the results and their real-world application.

The use of antiglaucoma medications at 12 months was significantly decreased in our microshunt patients (0.7±0.1), compared with preoperatively (3.4±0.1). In the recent RCT, the microshunt group had a similar mean number of anti-glaucoma medications (0.6±1.1) at 1 year.^12^ Durr *et al* also described a significant reduction in the use of medications, with 64.5% of their patients becoming medication free by 12 months.^8^ These results for the SIBS microshunt are promising and appear to reflect those described by several studies for the ab interno Xen gel implants.^21–23^ Likewise, the results for the SIBS microshunt are similar, if not better, than those described by the Tube Versus Trabeculectomy (TVT) study for Baerveldt tubes and trabeculectomies.^15^ The number of anti-glaucoma medications at 12 months were 2.1±1.4 in the Baerveldt tube group and 0.9±1.4 in the trabeculectomy group.^15^

In the TVT study, 41% of patients in the trabeculectomy group (N=48) and 29% in the baerveldt tube group (N=36) developed complications at 12 months.^15^ However, serious complications that required reoperation or produced a loss of 2 or more Snellen lines of vision occurred in only 8% of the trabeculectomy group and 1% of the Baerveldt tube group. In our study, 11.5% required an ab externo bleb revision and 1.9% required further glaucoma surgery at 1 year. Hypotony was also reported in 19.2% in our cohort in the early postoperative period, which was lower than the 28.9% and 49.6% in the microshunt and trabeculectomy groups, respectively, in the RCT.^12^

Antimetabolites, such as 5-FU and MMC, are commonly used both intraoperatively and postoperatively to supress fibroblast activity and hence the deposition of fibrotic tissue, which can lead to the obstruction of stents and failure of glaucoma filtration surgery. In our study, bleb encapsulation was reported in 1.9% and 4.8% in the early and late postoperative periods, respectively. Single and repeated 5-FU injections were given in 33.7% and 24.0% at 12 months, respectively. Needling was also performed in 12.5% at 12 months and was similar to the needling rates reported in other microshunt studies (11.8%, 8.5%),^8 11^ but slightly lower than the rate reported in the RCT (19.0%).^12^

As well as efficacy, the initial and long-term costs of the Preserflo microshunt are also important to consider. The SIGHT study (http://www.clinicaltrials.gov; ClinicalTrials.gov identifier: NCT01881425) investigating the cost-effectiveness of the InnFocus implant versus Trabeculectomy is underway in the Netherlands; however, the results will not be available until July 2022. The Preserflo microshunt costs £820 in the UK and the cost could be offset by a lower number of follow-up visits and early interventions (only 2.2% required an intervention at day 1 in our study), as well as a low rate of revision surgeries (only 11.5% required bleb revision in the first year). Cost-effectiveness studies of the Preserflo microshunt are warranted, but the reduced rates of early post-operative visits, interventions, and return to theatre might make it more cost-effective than other types of glaucoma surgeries.

Our study contributes real-world and generalisable data on the surgical outcomes of the PreserFlo MicroShunt that would help in clinical decision making in glaucoma surgery. The limitations are that it is a retrospective multicentre study and there were variations in the duration of MMC treatment intraoperatively. There was also a lack of standardisation for postoperative management, including when to inject 5-FU or steroids, when to perform needling procedures, and the criteria for restarting antiglaucoma medications.

In summary, the PreserFlo MicroShunt with MMC 0.4 mg/mL showed a lower success rate at 1 year compared with published trabeculectomy figures, but a slightly higher success rate compared with the RCT using MMC 0.2 mg/mL. However, this is a new device with a learning curve for surgeons, which can affect comparison with the more established trabeculectomy surgery. Nevertheless, the PreserFlo MicroShunt led to significant IOP and medication reduction at 1 year in our three UK tertiary centres, and exhibited a good safety profile with a low rate of adverse effects.

## Data Availability

All data relevant to the study are included in the article or uploaded as online supplemental information. Not applicable.
