# Supplementary material for: One-year surgical outcomes of the PreserFlo MicroShunt in glaucoma: a multicentre analysis
Source: Br J Ophthalmol. 2022 Apr 1;107(8):1104–11. doi: 10.1136/bjophthalmol-2021-320631 (PMC10359526; doi:10.1136/bjophthalmol-2021-320631)
Supplement: Supplementary data [file bjophthalmol-2021-320631supp001.pdf]

Supplementary Table 1. Number of anti-glaucoma medications by reasons for treatment failure at 1 year

Reasons for failure	Number of anti-glaucoma medications					
	**0**	**1**	**2**	**3**	**4**	**5**	**Total**
A* IOP > 21 mmHg as isolated cause	0	1	0	0	0	0	1
B* <20% IOP reduction as isolated cause	7	2	1	1	0	0	11
(A+B)* IOP > 21 and <20% IOP reduction (total 6)	3	2	1	0	0	0	6
**Total A* + B* + (A+B)***	**10**	**5**	**2**	**1**	**0**	**0**	**18**

Tanner A, *et al. Br J Ophthalmol* 2023; 107:1104–1111. doi: 10.1136/bjophthalmol-2021-320631
