# Supplementary material for: One-year surgical outcomes of the PreserFlo MicroShunt in glaucoma: a multicentre analysis
Source: Br J Ophthalmol. 2022 Apr 1;107(8):1104–11. doi: 10.1136/bjophthalmol-2021-320631 (PMC10359526; doi:10.1136/bjophthalmol-2021-320631)
Supplement: Supplementary data [file bjophthalmol-2021-320631supp002.pdf]

Supplementary Table 2. Summary of published papers on the PreserFlo^{TM} Microshunt

Study	Design	Number of eyes	Pre-op IOP	Post-op IOP	Pre-op visual field MD	Pre & post-op number of antiglaucoma drops	Complications, Interventions, Reoperations	MMC concentration	Complete success (CS) Qualified Success (QS)
Durr 2022[1]	Retrospective, single-centre series over 4 years looking at 1-year results of Microshunt implantation in cases of previously failed filtration surgery.	85 eyes from 79 patients	Median: 22.0	Median: 13.0	Mean: -15.4	Mean:<br>Preop = 4<br>Post-op = 0	**Choroidal detachment** 12.9%<br>**Hypotony maculopathy** 3.5%<br>**Early needling** 5.9%<br>**Late needling** 5.9%<br>**Reoperation** 7.1%	0.2-0.5 mg/ml for 2 minutes	**CS** 61.0%<br>**QS** 79.7%
Baker 2021[2]	Prospective, single-masked, multicentre, non-inferiority RCT for mild to severe POAG uncontrolled on maximal medical therapy. Randomised 3:1 Microshunt to trabeculectomy, 1-year results from 2-year study.	527 eyes (395 eyes Microshunt, 132 eyes trabeculectomy)	Mean:<br>Microshunt 21.1 ± 4.9<br>Trabeculectomy 21.1 ± 5.0	Mean reduction:<br>Microshunt 14.3 ± 4.3 (29.1%)<br>Trabeculectomy 11.1 ± 4.3 (45.4%)	44% moderate or severe disease,<br>MD < -12.01	Mean at 1 year:<br>Microshunt 0.6 ± 1.1<br>Trabeculectomy 0.3 ± 0.9	**At least 1 intervention**<br>Microshunt 40.8%<br>Trabeculectomy 67.4%<br>**Needling**<br>Microshunt 19%<br>Trabeculectomy 8.3%<br>**Transient hypotony**<br>Microshunt 28.9%<br>Trabeculectomy 49.6%<br>**Persistent hypotony**<br>Microshunt 7.7.1%<br>Trabeculectomy 21.2%<br>**Reoperation**<br>Microshunt 14.9%<br>Trabeculectomy 11.4%	0.2 mg/ml for 2 minutes	**Primary end point success**:<br>Microshunt 53.9%<br>Trabeculectomy 72.7%
Battle 2016[3]	Prospective, non-randomised, single-site study in patients with POAG progressing on maximal medical therapy without previous glaucoma surgery. Observations were made over 3 years.	23 eyes at 1 year, 22 eyes thereafter	Mean:<br>23.8 ± 5.3	Mean reduction:<br>1 year<br>10.7 ± 2.8 (55%)<br>2 years<br>11.9 ± 3.7 (50%)<br>3 years<br>10.7 ± 3.5 (55%)	Mean:<br>-20.1 ± 12.2<br>Range:<br>-2.4 to -33.9	Preop: 2.4 ± 0.9<br><br>Post op:<br>1 year 0.3 ± 0.8<br>2 years 0.4 ± 1.0<br>3 years 0.7 ± 1.1<br>71% mean reduction	**Choroidal detachment** 8.7%<br>**Transient hypotony** 13%<br>**Needling** 4.3%<br>**Reoperation** 4.3%	0.4 mg/ml for 3 minutes	**CS**<br>1 year 87%<br>2 years 86%<br>3 years 64%<br>**QS**<br>1 year 100%<br>2 years 100%<br>3 years 95%
Schlenker 2020[4]	Retrospective, single-centre case series of isolated Microshunt in open angle glaucoma where there has been no	164 eyes in 132 patients	Median: 20.0	Median: 12	Median: -11.4	Median:<br>Preop = 4<br>Postop = 0	**Choroidal detachment** 6.7% early, 2.5% late<br>**AC reformation** 3%<br>**Needling** 8.5%<br>**Reoperation** 1.8%	0.2-0.5 mg/ml for 2 minutes. Later cases received higher doses	Primary (IOP≤17mmHg):<br>**CS** 76.9%<br>**QS** 92.5%

	previous filtering or suprachoroidal surgery. 97% of eyes had 1 year follow up.								Secondary (IOP≤21mmHg):<br>**CS** 76.9%<br>**QS** 92.5%
Beckers 2021[5]	Prospective, single-arm, multicentre study over 2 years of standalone Microshunt in mild to moderate POAG that had not undergone prior incisional surgery.	81 eyes	Mean:<br>21.7 ± 3.4<br>Median:<br>20	Mean:<br>Year 1<br>14.5 ± 4.6<br>Year 2<br>14.1 ± 3.2	Not provided	Preop:<br>Mean 2.0 ± 1.3<br>Median 2.0<br><br>Postop:<br>Mean 0.5 ± 0.9	**Transient hypotony** 11.1%<br>**Needling** 6.2%<br>**Reoperation** 9.9%	0.2-0.4 mg/ml for 2 or 3 minutes	1 year:<br>**CS** 58.0%<br>**QS** 74.1%<br>2 years:<br>**CS** 59.3%<br>**QS** 74.1%
Scheres 2021[6]	Retrospective, comparative case series comparing Xen45 implantation to Microshunt in progressive POAG. Follow-up of at least 6 months with mean of 22.4 and 18.9 months, respectively. Combined cataract surgery was permitted.	82 eyes<br>(Xen: 41 eyes of 31 patients, Microshunt: 41 eyes of 33 patients)	Mean:<br>Xen45<br>19.2 ± 4.4<br>MicroShunt<br>20.1 ± 5.0	Mean:<br>Xen45<br>1 year<br>13.3 ± 2.9<br>2 year<br>13.8 ± 2.8<br><br>Microshunt<br>1 and 2 years<br>12.1 ± 3.5	71% moderate or severe disease,<br>MD < -12.01<br>Microshunt	Preop:<br>Xen45<br>2.5 ± 1.4<br>Microshunt<br>2.3 ± 1.5<br><br>Postop:<br>2 years<br>Xen45<br>0.9 ± 1.2<br>Microshunt<br>0.7 ± 1.1	Xen:Microshunt<br>**Early hypotony** 24:39%<br>**Late hypotony** 8:0%<br>**Needling** 20:5%<br>**Bleb revision** 5:5%<br>**Operative stent adjustment** 2:2%<br>**Filtration surgery** 7:15%	0.2 mg/ml for 3 minutes for Microshunt	Primary endpoint (IOP ≤18 mmHg), Xen:Microshunt<br>1 year:<br>**CS** 46:58%<br>**QS** 78:79%<br>2 years:<br>**CS** 34:49%<br>**QS** 73:79%
Nobl 2021[7]	Retrospective, single centre, interventional study of PXF glaucoma and POAG. 1-year results. Standalone or in combination with cataract extraction. Prior surgery including incisional filtering surgery was not excluded.	46 eyes of 41 patients<br>(PEXG 20 eyes, POAG 26 eyes)	PEXG<br>21.4 ± 5.8<br>POAG<br>18.2 ± 4.5	PEXG<br>12.8 ± 3.0<br>POAG<br>12.9 ± 4.2	PEXG<br>-7.65 ± 5.59<br>POAG<br>- 8.97 ± 7.12	Preop:<br>PEXG<br>2.8 ± 1.3<br>POAG<br>2.7 ± 1.3<br><br>Postop:<br>PEXG<br>0.3 ± 0.8<br>POAG<br>0.3 ± 0.8	**Hypotony**<br>PEXG 40.0%<br>POAG 11.5%<br>**Choroidal detachment**<br>PEXG 30.0%<br>POAG 3.8%<br>**AC reformation**<br>PEXG 10%<br>POAG 3.8%<br>**Reoperation**<br>PEXG 15.0%<br>POAG 7.7%	0.2 mg/ml for 2 minutes	Success criteria (IOP < 18 mmHg, >20% reduction):<br>PEXG<br>**CS** 75.0%<br>**QS** 80.0%<br><br>POAG<br>**CS** 73.1%<br>**QS** 76.9%
Pillunat 2021[8]	Prospective, interventional, cohort study comparing Microshunt to trabeculectomy in POAG eyes without previous	52 eyes<br>(Microshunt 26 eyes, Trabeculectomy 26 eyes)	Mean diurnal:<br>Microshunt<br>15.9<br>Trabeculectomy<br>17.1<br><br>Mean peak:	Mean diurnal:<br>Microshunt<br>10.8<br>Trabeculectomy<br>10.3<br><br>Mean peak:	Mean:<br>Microshunt<br>-8.7<br>Trabeculectomy<br>-12.9	Preop:<br>Microshunt<br>4<br>Trabeculectomy<br>4	Microshunt: Trabeculectomy<br>**Early hypotony** 69:27%<br>**Late hypotony** 0:8%<br>**AC reformation** 15:23%<br>**Choroidal detachment**	0.2 mg/ml for 3 minutes	Mild glaucoma, non-fixation threatening:<br>Microshunt<br>**CS** 100%<br>Trabeculectomy<br>**CS** 100%

	filtration surgery. Results at 6 months.<br>Success criteria: mean diurnal IOP ≤18 mmHg in glaucoma, non-fixation threatening, and ≤14 mmHg for mild glaucoma, fixation-threatening (diurnal peak IOP ≤18 mmHg), moderate or advanced glaucoma.		Microshunt<br>20<br>Trabeculectomy<br>22	Microshunt<br>13<br>Trabeculectomy<br>12.5			15:20%<br>**Needling** 4:27%<br>**Reoperation** not recorded		Mild glaucoma fixation-threatening, moderate or advanced glaucoma:<br>Microshunt<br>**CS** 90%<br>**QS**95%<br>Trabeculectomy<br>**CS** 87%<br>**QS** 87%
Quaranta 2021[9]	Retrospective review across 2 centres in Italy of Microshunt in POAG following failed primary trabeculectomy. 12-month follow-up.	31 eyes	Mean:<br>24.8 ± 3.86	Mean:<br>12.56 ± 2.64	Mean:<br>-6.17 ± 1.88	Preop:<br>3.3 ± 0.6<br>Postop:<br>0.5 ± 0.8	**Transient hypotony** 19.3%<br>**Choroidal detachment** 9.6%<br>**Needling** 19.3%<br>**Reoperation** 3.2%	0.3 mg/ml for 3 minutes	Success (IOP ≤ 17 mmHg):<br>**CS** 67.74%<br>**QS** 93.54%
Martinez-de-la-casa 2021[10]	Retrospective, 2-centre, open-label study of Microshunt as standalone or combined-with-phaco procedure in uncontrolled open angle glaucoma with minimum 12-month follow-up data.	58 eyes (Microshunt standalone 35 eyes, combined phaco 23 eyes)	Mean:<br>Microshunt<br>21.3 ± 3.2,<br>Combined phaco<br>21.5 ± 3.3	Mean:<br>Microshunt<br>14.4 ± 3.4,<br>Combined phaco<br>14.9 ± 3.6	Mean:<br>-6.2 ± 3.9	Preop:<br>2.3 ± 0.5<br>Post op:<br>0.2 ± 0.5	**Hypotony** 1.7%<br>**Choroidal detachment** 3.5%<br>**Needling** not recorded<br>**Reoperation** 0	0.2 mg/ml for 2 minutes	Success (IOP ≤ 18 mmHg):<br>**CS** 62.1%<br>**QS** 82.8%
Vastardis 2021[11]	Retrospective, single-site study of Microshunt in refractory, moderate to advanced POAG in pseudophakic eyes. Implantation was with MMC, with and without Ologen collagen matrix (OCM). No previous filtering or conjunctival surgery.	50 eyes<br>(25 eyes with OCM, 25 eyes without OCM)	Mean:<br>Without OCM<br>23.5 ± 5.8<br>With OCM<br>26.0 ± 8.8	Mean:<br>Without OCM<br>11.6 ± 3.1<br>With OCM<br>11.8 ± 3.4	Mean:<br>Without OCM<br>-13.15 ± 8.53<br>With OCM<br>-13.47 ± 7.44	Mean Preop:<br>Without OCM<br>2.5 ± 0.9<br>With OCM<br>2.6 ± 0.8<br><br>Mean Postop:<br>Without OCM<br>0.0 ± 0.2<br>With OCM<br>0.2 ± 0.8	**Early hypotony** 24%<br>**Choroidal detachment** 14%<br>**Needling** not recorded<br>**Reoperation** 8% (1 case no OCM**,** 3 cases OCM)	0.2 mg/ml for 3 minutes	Success (IOP ≤ 21 mmHg):<br>Without OCM<br>**CS** 68%<br>**QS** 92%<br><br>With OCM<br>**CS** 58.3%<br>**QS** 95.8%

References

1. Durr GM, Schlenker MB, Samet S, et al. One-year outcomes of stand-alone ab externo SIBS microshunt implantation in refractory glaucoma. *Br J Ophthalmol.* 2022;106(1):71-79.
2. Baker ND, Barnebey HS, Moster MR, et al. Ab-externo MicroShunt versus Trabeculectomy in Primary Open-Angle Glaucoma: 1-year Results from a 2-year Randomized, Multicenter Study. *Ophthalmology.* 2021;128(12):1710-1721.
3. Batlle JF, Fantes F, Riss I, et al. Three-Year Follow-up of a Novel Aqueous Humor MicroShunt. *J Glaucoma.* 2016;25(2):e58-65.
4. Schlenker MB, Durr GM, Michaelov E, et al. Intermediate Outcomes of a Novel Standalone Ab Externo SIBS Microshunt With Mitomycin C. *Am J Ophthalmol.* 2020;215:141-153.
5. Beckers HJM, Aptel F, Webers CAB, et al. Safety and Effectiveness of the PRESERFLO® MicroShunt in Primary Open-Angle Glaucoma: Results from a 2-Year Multicenter Study. *Ophthalmol Glaucoma.* 2021 Jul 28:S2589-4196(2521)00179-00174.
6. Scheres LMJ, Kujovic-Aleksov S, Ramdas WD, et al. XEN Gel Stent compared to PRESERFLO™ MicroShunt implantation for primary open-angle glaucoma: two-year results. *Acta Ophthalmol.* 2021;99(3):e433-e440.
7. Nobl M, Freissinger S, Kassumeh S, et al. One-year outcomes of microshunt implantation in pseudoexfoliation glaucoma. *PLoS One.* 2021;16(8):e0256670.
8. Pillunat KR, Herber R, Haase MA, et al. PRESERFLO™ MicroShunt versus trabeculectomy: first results on efficacy and safety. *Acta Ophthalmol.* 2021 Jul 31:doi: 10.1111/aos.14968.
9. Quaranta L, Micheletti E, Carassa R, et al. Efficacy and Safety of PreserFlo MicroShunt After a Failed Trabeculectomy in Eyes with Primary Open-Angle Glaucoma: A Retrospective Study. *Adv Ther*. 2021;38(8):4403-4412.
10. Martínez-de-la-Casa JM, Saenz-Francés F, Morales-Fernandez L, et al. Clinical outcomes of combined Preserflo Microshunt implantation and cataract surgery in open-angle glaucoma patients. *Sci Rep*. 2021;11(1):15600.
11. Vastardis I, Fili S, Perdikakis G, et al. Preliminary results of Preserflo Microshunt versus Preserflo Microshunt and Ologen implantation. *Eye Vis (Lond)*. 2021;8(1):33.
